# Protocol for a randomised controlled trial of telemonitoring and self-management in the control of hypertension: Telemonitoring and self-management in hypertension. [ISRCTN17585681]

**DOI:** 10.1186/1471-2261-9-6

**Published:** 2009-02-16

**Authors:** Richard J McManus, Emma P Bray, Jonathan Mant, Roger Holder, Sheila Greenfield, Stirling Bryan, Miren I Jones, Paul Little, Bryan Williams, FD Richard Hobbs

**Affiliations:** 1 Primary Care Clinical Sciences, Primary Care and Clinical Sciences Building, University of Birmingham, Birmingham, UK; 2 General Practice and Primary Care Research Unit, University of Cambridge, Cambridge, UK; 3 Centre for Clinical Epidemiology & Evaluation, Vancouver Coastal Health Research Institute, Vancouver, Canada; 4 Community Clinical Sciences, University of Southampton, Southampton, UK; 5 Department of Cardiovascular Sciences, University of Leicester, Leicester, UK

## Abstract

**Background:**

Controlling blood pressure with drugs is a key aspect of cardiovascular disease prevention, but until recently has been the sole preserve of health professionals. Self-management of hypertension is an under researched area in which potential benefits for both patients and professionals are great.

**Methods and design:**

The telemonitoring and self-management in hypertension trial (TASMINH2) will be a primary care based randomised controlled trial with embedded economic and qualitative analyses in order to evaluate the costs and effects of increasing patient involvement in blood pressure management, specifically with respect to home monitoring and self titration of antihypertensive medication compared to usual care. Provision of remote monitoring results to participating practices will ensure that practice staff are able to engage with self management and provide assistance where required. 478 patients will be recruited from general practices in the West Midlands, which is sufficient to detect clinically significant differences in systolic blood pressure between self-management and usual care of 5 mmHg with 90% power. Patients will be excluded if they demonstrate an inability to self monitor, their blood pressure is below 140/90 or above 200/100, they are on three or more antihypertensive medications, have a terminal disease or their blood pressure is not managed by their general practitioner.

The primary end point is change in mean systolic blood pressure (mmHg) between baseline and each follow up point (6 months and 12 months). Secondary outcomes will include change in mean diastolic blood pressure, costs, adverse events, health behaviours, illness perceptions, beliefs about medication, medication compliance and anxiety. Modelling will evaluate the impact of costs and effects on a system wide basis. The qualitative analysis will draw upon the views of users, informal carers and professionals regarding the acceptability of self-management and prerequisites for future widespread implementation should the trial show this approach to be efficacious.

**Discussion:**

The TASMINH2 trial will provide important new evidence regarding the costs and effects of self monitoring with telemonitoring in a representative primary care hypertensive population.

**Trial Registration:**

ISRCTN17585681

## Background

National and international surveys have found that despite some recent improvements, the blood pressure of many people with hypertension remains poorly controlled. [1,2] This is despite evidence that reduction to <140/90 mmHg is achievable and results in important benefits in terms of morbidity and mortality.[3] Adequate blood pressure control is a prerequisite if cardiovascular disease is to be reduced in line with recent UK policy targets.[4,5]

There are many potential reasons for sub-optimal blood pressure control. Hypertension is largely asymptomatic with little biological feedback to patients in terms of blood pressure control. Treatment is generally life long and may be associated with dose related side effects. It is therefore perhaps not surprising that adherence to antihypertensive medication is often poor with around half of hypertensive patients discontinuing new prescriptions for antihypertensive drugs in the first six months.[6]

It is not simply a question of motivating patients. Research in UK general practice has shown a lack of professional action in the presence of high recorded blood pressures along with a reluctance to prescribe for isolated systolic hypertension.[7] Fear of side effects may be one reason for this inaction although modern treatment trials have shown relatively modest levels of side effects.[2] Workload in primary care has been cited as another reason for poor performance, with the average practice having to provide care for several hundred people with hypertension.[8] Novel approaches for the management of high blood pressure are therefore required.

There is good evidence for both pharmacological and non-pharmacological interventions.[9] The advent of accurate and easy to use automated sphygmomanometers means that blood pressure measurement need no longer be confined to professionals. Health behaviour models suggest that increased patient involvement in disease management will result in cues to action and increased self-efficacy with improved adherence to treatment and beneficial changes in other health behaviours such as in diet and exercise.[10]

Four previous randomised studies have evaluated the efficacy of telemonitoring in conjunction with self-monitoring of hypertension and other interventions but none have been adequately powered, followed up for more than six months, or set in the UK. [11-14](Table 1) Friedman's 1996 study randomised 267 hypertensive patients under the care of community physicians to self-monitoring with an interactive telephone reporting system or usual care. Intervention patients measured their own blood pressure on a weekly basis and reported the results via the "TLC" computerised telephone system. [11] This system, as well as recording blood pressure, provided automated feedback to the subject before transmitting results to an individual's physician. The authors found a significant benefit from the intervention in terms of diastolic blood pressure once adjusted for baseline differences between the groups. No such difference was seen for systolic blood pressure. Roger and colleagues studied 121 hypertensive people recruited from a hospital clinic. [12] Automated blood pressure readings were transmitted to the study centre via telephone. The intervention group had slightly reduced (3 mmHg) mean arterial pressure as measured by twenty-four hour blood pressure monitoring which remained significant after adjustments for baseline differences between groups. The final two trials by Mehos (34 patients) [13] and Artinian (26 patients) [14] were too small to draw conclusions from other than the fact that community based telemonitoring of blood pressure is feasible in a range of settings.

**Table 1:** Summary of randomised studies evaluating self-monitoring with telemonitoring or self-management

**Study**	**Number of subjects** **Mean age** **Length of follow up**	**Type & frequency of BP self measurement**	**Frequency of other input (either as co-intervention or outcome measurement)**	**Was physician adjusting medication aware of self measurement readings**	**Outcome measurement**	**Outcome of study**
Friedman 1996 US [11]	267 patients under care of community physicians Age 77 6 months	Automated Weekly (?upper arm)	Self report of BP, adherence etc via computerised telephone system (TLC) on a weekly basis with automated feedback	"TLC" data transmitted to patient's own physician	BP measured on home visit; protocol for measurement not clear if blinded	Small drop in DBP only after adjustment (mean adjusted DBP change 5.2 mmHg vs 0.8 mmHg, No CIs, p = 0.02)
Mehos [13] 2000 US	36 primary care patients with poorly controlled hypertension 59 years 6 months	Manual electronic Daily Upper arm	Monthly telephone calls to coordinate treatment changes All received counselling on ht rx and lifestyle	Yes	not clear if blinded	No CI s or p quoted
Rogers [12] 2001 US	121 hypertensive patients from hospital clinic 61 years At least 8 weeks	Automated 3 days per week (? upper arm)	Transmission of results of BP down phone line Monthly reports to patient and physician electronically generated	Yes	Main outcome ambulatory BP monitoring pre and post intervention which physicians were blinded to	Reduction in MAP of 3 mmHg, (no CI, p = 0.013)
Artinian [14] 2001 US	26 African American with hypertension attending a family community centre	Automated At least 3 days per week Upper arm	Transmission of BP results down phone line each Friday with automated feedback to patients Additional feedback via study nurse	Yes	Clinic measurement before and after measured by blinded investigator	Pilot study: no formal comparison of between group BP drop.
Zarnke [15] 1997 Canada	31 hypertensive primary care patients 55 years 8 weeks	Electronic Twice daily (?upper arm)	Self directed adjustment of medication Self help advice to all	Yes if consulted when patient had already tried to change treatment	Mercury sphyg; not clear if blinded but externally measured. Also ambulatory BP	Intervention group had lower mean ambulatory MAP.(-0.95 vs +1.9 mmHg, No CI, p = 0.039)

A further study [15] by Zarnke and colleagues in Canada has evaluated self-management of hypertension (Table 1). 31 patients with hypertension were randomised between usual care and self-directed adjustment of medication using a fixed set of antihypertensive medication. Follow-up after eight weeks showed a small drop in mean arterial pressure in the intervention group compared to control.

These studies have been combined with other self-monitoring work in two systematic reviews. [16,17] These showed that, in common with other non-pharmacological interventions, self-monitoring, with or without telemonitoring, has a modest effect on blood pressure. One subsequent randomised study has evaluated self-monitoring in a UK primary care setting. [18] This work by our group showed that people with hypertension were able and willing to self-monitor blood pressure, and that the small reductions in blood pressure were probably mediated through lifestyle changes and that self-monitoring is cost effective.

The studies discussed above have all been quantitative studies and there has been very limited exploration of patients' views of self-monitoring. [19,20] There is currently no information on potential issues arising from self-management of hypertension from the perspective of patients, carers or health care providers.

Therefore, the TASMINH2 study is an extension of our previous work, expanding the intervention from self monitoring to self management to include not only self monitoring of blood pressure, but telemonitoring and self titration of antihypertensive medication, with the expectation that this will result in larger reductions of blood pressure.

## Methods

### Study Aims, Research Questions, and Outcomes

The primary aim of TASMINH2 is to compare self management with usual care in the control of hypertension. The trial has four main research questions:

1) Does self-management with telemonitoring and titration of antihypertensive medication by people with poorly controlled hypertension result in better control of blood pressure?

2) Is self-management with telemonitoring and titration of antihypertensive medication by people with poorly controlled hypertension cost effective?

3) Is self-management with telemonitoring and titration of antihypertensive medication achievable in routine practice and is it acceptable to patients?

4) What are the views and experiences of patients, informal carers and healthcare professionals of self-management with telemonitoring and titration of antihypertensive medication?

The primary outcome of the trial will be the change in mean systolic blood pressure (mmHg) between baseline and each follow up point (6 months and 12 months), measured in the surgery by the research team. Additional secondary outcomes will include change in mean diastolic blood pressure, adverse events (side effects, anxiety), health behaviours, illness perceptions, beliefs about medication, medication compliance, patient satisfaction, costs and reasons for non-participation as well as the qualitative analysis and health economic modelling beyond the trial outcomes.

### Study Design and Setting

TASMINH2 is a primary care based, unblinded, randomised controlled trial with embedded economic and qualitative analyses in order to evaluate the costs and effects of increasing patient involvement in blood pressure management.

### Ethical considerations

Full ethical approval for this trial has been obtained from Sandwell and West Birmingham local ethics committee (reference; 05/Q2709/103), whilst site specific ethical approval and R & D approval was obtained from the relevant local ethics committees and Primary Care Trusts. A trial steering group will monitor study progress.

### Trial Interventions

Usual care will consist of the participant seeing their General Practitioner (GP) (Family Physician) and/or nurse periodically for blood pressure measurement and/or adjustment of medication at the discretion of the GP.

Self-management will consist of self-monitoring of blood pressure with electronic transmission of readings, and self titration of medication dependant on the self-monitoring readings.

#### Blood pressure self-monitoring

Participants will be trained to monitor their blood pressure using an automated electronic sphygmomanometer. Home readings will be transmitted to the research team via the telemonitoring system. Patients will self-monitor blood pressure daily for the first week of each month of the study. They will be provided with a guideline that contains simple colour coded instructions. Very high or very low readings will require checking by the participant's practice. Four or more above target readings in two consecutive months will require a change in medication. Below target readings will simply require further monitoring the following month.

#### Target Blood Pressure

Target blood pressures will be based on the NICE hypertension guideline with adjustment of 10/5 mmHg as recommended by the British Hypertension Society to reflect home as compared to office readings. [21,22] People without diabetes will have a home target of ≤130/85 mmHg. People with diabetes or CKD stage 3–5 will have target blood pressures of ≤130/75 mmHg to take into account the lower NICE recommendations.

#### Telemonitoring

Participants will be trained in the use of the telemonitoring equipment at baseline and home visits (practical technical help) will be available if required to set up equipment. The equipment consists of a standard validated automated electronic sphygmomanometer (Omron 705-IT) with a modem for telemonitoring (Melexis I-modem). Blood pressure data (including time and date) will be transferred to the research team from the memory of the electronic sphygmomanometer via an internet connection. Practices will receive summary data from the research team once each month, by report to allow incorporation into clinical records systems. In addition, blood pressure recordings will also be available to patients via a secure internet site which will summarise individual patient's monitoring results.

#### Self-titration of medication

Each participant will be given an individually tailored two step self management algorithm through which to adjust medication according to measured blood pressure. The choice of medication changes will be decided in conjunction with their own GP. Each step will represent a single medication change (additional medication or increased dose) that will be made following raised readings in two consecutive months. Medication choice will remain that of the GP who will be provided with a copy of the NICE hypertension management algorithm to aid choice of medication. If patients have used both steps of their self-management algorithm they will return to the GP and a new two step plan will be devised. As the study will last for 12 months, patients will not require any more than two such management plans. Any additional monitoring (for instance blood tests or urinalysis) will be the responsibility, and at the discretion, of the GP.

### Non-Participation

Included with the letter of invitation to take part in the trial, will be a form for people to voluntarily return should they wish to decline the invitation. As well as asking for their reasons for wishing to decline, this form will also seek permission to send a further questionnaire and/or gain their permission for the research team to have access to their medical records. The further questionnaire will be similar to that which will be used in the research clinics/follow-up sessions with people enrolled in the study. Following-up those who decline to take part will provide useful information regarding the generalisability of the trial results, as well as providing a greater insight into the reasons why people may not wish to self-manage their hypertension.

### Study Population, Sampling and Recruitment Procedure

The study population will comprise of people with poorly controlled treated hypertension managed in primary care. Eligibility criteria will be age between 35–85, treated hypertension, and blood pressure greater than 140/90. Exclusion criteria will be inability to self-monitor (including diagnosis of dementia, score of >10 on short orientation memory concentration test), postural hypotension (systolic blood pressure drop >20 mmHg), more than two antihypertensive medications, terminal disease, and blood pressure not managed by their GP. (see Table 2)

**Table 2:** Inclusion and Exclusion Criteria

**Inclusion Criteria**
Age between 35–85
Treated hypertension (diagnostic code for hypertension plus prescription for antihypertensive medication)
Blood pressure greater than 140/90 at baseline
Willingness to self monitor and self manage
**Exclusion Criteria**
Inability to self monitor (including diagnosis of dementia, score of >10 on short orientation memory concentration test)
Current prescription for more than two antihypertensive medications
Terminal disease
Blood pressure not managed by their General Practitioner
Postural Drop > 20 mmHg systolic

Eligible patients will be identified from around 15 general practices in the West Midlands area drawn from the Midlands Research Practices Consortium (MidReC).[23] A member of the research team will conduct a practice-based computer search to identify patients that fulfil the eligibility criteria. General Practitioners will be asked to check these computer generated lists to remove people who are known to have terminal illness, those not managed by the GP or who are thought by the GP to be unsuitable for the study. Previous experience from the TASMINH trial suggests that around 25–30% of people receiving treatment for hypertension will take part in a study such as that proposed.[18] We plan to recruit patients from each practice over a 2–3 week period before moving on to the next practice. Assuming 40 patients per practice are recruited then around 50 will need to be screened and so at least 200 will need to be invited.

Participants who withdraw from the trial will not be replaced, but asked if they are prepared to continue to attend follow-up clinics. Analysis will be by intention to treat.

### Randomisation

Randomisation of patients with uncontrolled hypertension will be to either usual care or self-management of their hypertension and will take place centrally via telephone or internet using the process of minimisation taking into account practice, sex, diabetic/chronic kidney disease status, baseline blood pressure and age.

### Study Clinics and Flow through Study

At baseline all patients will attend a clinic at which the study will be explained, informed consent gained and questionnaires regarding demographics, past medical history, self-efficacy and attitudinal compliance will be completed (Table 3). Measurement of blood pressure and BMI will also be performed and baseline economic data collected. Patients will be randomised to either usual care or self-management. People randomised to usual care will be asked to book an appointment approximately one week later for a medication review with their usual GP. People randomised to self-management will be asked to book an appointment for a group training session where they will be trained to use an electronic sphygmomanometer and the telemonitoring system. After the group training session, patients will be asked to practice at home using their blood pressure monitors and telemonitoring system, and return approximately one week later for an individual training session which will cover the self-titration part of the intervention. If necessary a third training session will be included for additional support. Following satisfactory training (assessed by one of the research team), patients will be asked to make an appointment with their GP to establish a two step management plan for any potential medication changes. Patients not judged to be able to self-manage will be given the option to self-monitor alone.

**Table 3:** Data Collection at Study Clinics

Baseline Only:
1. Demographic questions; including race, occupation, marital status, employment, education,
2. Length of hypertension
3. Past medical history
4. Contraindications/intolerance to antihypertensives
5. Short orientation memory concentration test[29]
6. Height
7. Current self monitoring behaviour

Baseline and subsequent follow up
1. Current antihypertensive medications
2. Symptom section of the Illness Perception Questionnaire [30]
3. Weight
4. New medical history (in last 6 months)
5. Preference for blood pressure measurement methods
6. Beliefs About Medicines Questionnaire [31]
7. Medication Compliance sub-scale of the Hill-Bone Compliance to High Blood Pressure Medication Scale [32]
8. Short-form of the State-Trait Anxiety inventory [33]
9. EQ5-D [34]
10. Godin's Exercise Questionnaire [35]
11. Questions on smoking, alcohol consumption and salt intake
12. Economic data (referrals, consultations, resource use, willingness to pay)
13. Blood Pressure (sitting plus standing at baseline only)

**Figure 1:**
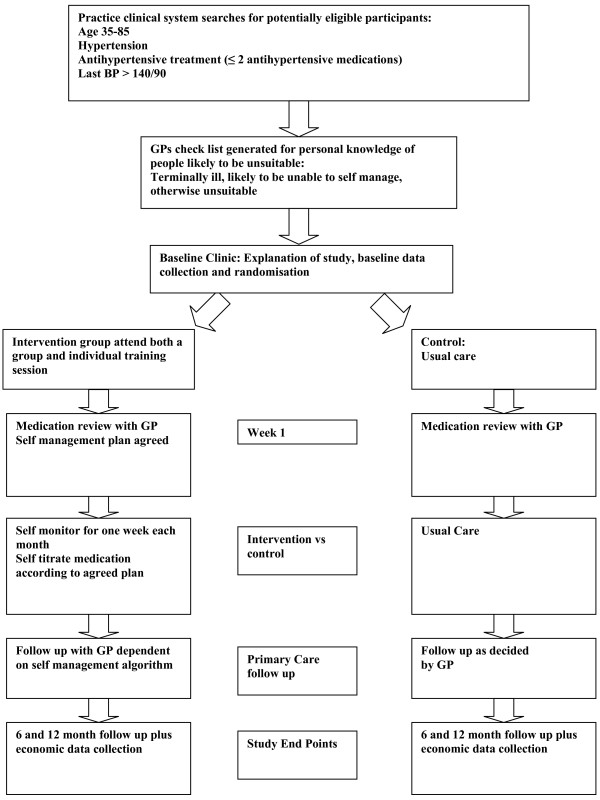
**Flow through the study**.

All patients will be asked to attend two follow-up clinics; one at 6 months and one at 12 months. Each clinic will be timetabled for approximately half an hour, in which time patients will have their blood pressure measured by the research team using a validated automated electronic sphygmomanometer, and will be asked to complete a questionnaire similar to that completed at baseline. Additionally, the research team will check that patients in the intervention arm are using the blood pressure monitors correctly. This is shown in Figure 1.

### Sample size considerations

A sample size of 191 people per group is required for 90% power assuming a standard deviation of 15 mmHg and a difference in reduction of blood pressure of at least 5 mmHg between intervention and control groups. A reduction of 5 mmHg systolic blood pressure is plausible in comparison with other studies of self-monitoring of blood pressure and should result in around 19% reduction in stroke risk. Clinic measurements are used as the primary end point as these are comparable to most treatment studies. Based on the drop out rate from the initial TASMINH self monitoring trial, and taking into account the additional requirements of this trial, a 20% drop out rate during follow-up is assumed, meaning that a sample size of 239 per group will need to be randomised, i.e., 478 people. We estimate that between 10–15 practices will be required i.e., 35–50 patients per practice.

### Statistical Analysis

Analysis will be on an intention to treat basis for complete cases. A sensitivity analysis will examine the potential effect of missing data. This will include replacement of missing data by the most recent previous data or by the mean of the series. The principal analysis will be performed using both raw and adjusted data and will be performed at the end of the trial after all data has been collected. The primary analysis will be using general linear modelling (GLM) to compare intervention and control systolic blood pressure at follow-up adjusting for baseline blood pressure, practice (as a random effect), diabetic or CKD status and sex. Planned sub group analyses will be of diabetic vs non-diabetic patients, older vs younger (65 as threshold), males vs females, better controlled at baseline vs worse controlled at baseline (> vs < 155 mmHg systolic).

### Economic analysis

The cost analysis will include all key resources associated with the self-management intervention and related health and social care services, and will adopt a broad perspective to include NHS, social care and patient costs. The implication of this being that we will measure the basic set-up and delivery costs of the self-management package, in addition to costs falling on other health and social care agencies and the research participants themselves. Health sector cost data (consultations, medications, referrals) will be collected prospectively by routine practice computer systems and downloaded by the research team at the final follow up visit. Intervention costs, including equipment and training, will be collected by the research team. Patient costs and broader social care resource use will be collected using a modified version of a patient cost questionnaire used successfully by the researchers in other settings. Unit cost data will be ascertained for consultation and referral costs for PCT and system modelling. Drug costs will be extracted from a contemporaneous BNF.

The economic analysis will involve use of cost-consequences analysis, [24] whereby important aspects of costs and benefits will be analysed and described in their natural units (e.g., incidence of stroke or diabetes complications). In addition, the use of the EQ-5D instrument in the battery of outcome measures also allows a within-trial cost-utility analysis (CUA) to be undertaken, with benefits aggregated in terms of quality-adjusted life years (QALYs). The project will also have a modelling component, the main purpose of which is to allow for extrapolation beyond the observed outcomes (i.e. the use of a modelling framework provides the opportunity to predict longer-term outcomes based on the study results). Such longer-term outcomes include: patient survival, longer-term quality of life and the full costs associated with long-term events.

A cost benefit analysis will be performed using willingness-to-pay data collected through a postal survey towards the end of the trial. These approaches will allow us to capture in a quantitative manner the broader non-health benefits of self-management.

### Qualitative Sub Study

#### Qualitative participants and sampling

Purposive sampling will be used to choose patients (both intervention and control), informal carers and healthcare professionals (up to 20 of each) to allow a wide range of views and experiences of self-monitoring. This will be followed by seeking a further theoretical sample within each group with selection guided by emerging data analysis in order to extend and challenge earlier data and interpretation, and test the integrity and credibility of the developing analysis.[25] This theoretical sample may include individuals with particular experiences that it becomes important to seek as the analysis develops. It is anticipated that up to 10 further respondents from each of patients, carers and professionals may be sought in this way (maximum 90 participants), before interviewing is no longer generating new concepts i.e. theoretical saturation.[26]

### One to one interviews

Data from patients and informal carers will be collected by confidential, face-to-face interview in patients' own homes, and from healthcare professionals in their GP practice using an interview topic prompt.[27] Patients and informal carers will be interviewed after approximately 6 months in the study. The interviews will follow broad topic areas based upon the study objectives and previous experience of self-monitoring,[18] but encourage respondents to discuss their perceptions and experiences freely. Topics will include: previous experience of self-monitoring of hypertension; views on the monitoring equipment, training, written instructions and guidelines; concerns about ability to self-manage; concerns about adjusting medication; support from family and healthcare professionals during self-management; effect of self-management on daily life and lifestyle changes; benefits and problems with self-management; views on medication. Their experience of self-management either as user, informal carer or professional will be specifically focussed on and explored in depth. The acceptability of self-management programmes and any preference they initially had for standard care or self-management will be explored.

All interviews will be audio-taped and transcribed and entered into the NVivo software package for qualitative data analysis.

### Qualitative analysis

Constant comparative analysis will be used to interpret the data.[28] To maximise theoretical sensitivity, researchers from different disciplinary and professional backgrounds will contribute to the development of the analysis and conceptual framework.[26] Coding processes will be aided by application of the NVivo software in identifying emerging key categories and concepts from the data.[26] These will be compared across the different interview data sources and established concepts in the literature. Data collection and analysis will be iterative, occurring as data collection in the interviews proceeds with new data being used to challenge, assess or confirm the emerging analysis. Concepts identified will be integrated into themes providing a structure for presentation of findings.

## Discussion

The results of this trial will be directly applicable to primary care in the UK. As only half of the approximately 12% of the adult population who are receiving treatment for hypertension are controlled to recognised targets, should self management of hypertension with telemonitoring be found to be a successful strategy, then it would be applicable to many hundreds of thousands of individuals in the UK and beyond.

We anticipate that the potential risks of this study are low and similar to those attributable to usual care. Particular issues are where a patient finds an excessively high or low reading and the possibility of increased anxiety due to the study. The patient guideline will advise contact with the supervising physician or nurse in the case of excessively high or low readings. Training of participants will cover repeated measurements in the case of unusually high or low readings and a helpline will be available should subjects require advice over and above that available in the guideline. We will carefully collect data on adverse events.

## Competing interests

The authors declare that they have no competing interests.

## Authors' contributions

RM, JM, RHob, RHol, SG, SB, PL and BW were responsible for identifying the research question and contributing to the drafting of the study protocol. EB and MJ have contributed to the development of the protocol and study design as members of the research team. RM and EB were responsible for the drafting of this paper although all authors provided comments on the drafts and have read and approved the final version.

## Pre-publication history

The pre-publication history for this paper can be accessed here:


